# Saline Flush versus Chest x ray in Confirmation of Central Venous Catheter Placement; a Diagnostic Accuracy Study 

**Published:** 2017-08-08

**Authors:** Mehrdad Esmailian, Reza Azizkhani, Nazila Najafi

**Affiliations:** 1 Emergency Medicine Research Center, Department of Emergency Medicine, Al-Zahra Research Institute, Isfahan University of Medical Sciences, Isfahan, Iran.; 2 Emergency Department, Student Research Committee, Faculty of Medicine, Isfahan University of Medical Sciences, Isfahan, Iran.

**Keywords:** Catheterization, central venous, sensitivity and specificity, ultrasonography, interventional, diagnostic imaging, chest x ray

## Abstract

**Introduction:**

Central venous catheterization (CVC) is a commonly performed procedure in critically ill patients of emergency department. This study was designed to compare the diagnostic accuracy of saline flush with CXR in confirmation of above-the-diaphragm CVC placement.

**Methods:**

This prospective cross sectional study was conducted on adult patients in need of CVC placement in emergency department. Placement Confirmation was performed with saline flush method and CXR, then chest computed tomography (CT) was performed as the gold standard. The screening performance characteristics of the two methods were calculated and compared using SPSS 21 and STATA 11.

**Results:**

103 cases with the mean age of 57.18±9.3 (35 -80) years were studied (52.4% male). The mean duration of procedure was 2.5±1.24 in saline flush and 32.11±5.52 minutes in CXR method (P<0.001(. The area under the ROC curves for saline flush and CXR in confirmation of CVC placement were 0.90 (95%CI: 0.70 – 0.100) and 0.80 (95%CI: 0.55 – 0.100), respectively (p = 0.317). The sensitivity, specificity, positive and negative predictive value, and positive and negative likelihood ratio of saline flush were 80%, 100%, 100, 98.9%, Infinity, and 0.01, respectively. These measures were 60%, 100%, 100%, 98%, Infinity, and 0.02 for CXR, respectively.

**Conclusion:**

It seems that saline flush method could be considered as a safe, rapid, and accurate bedside method for CVC placement confirmation in emergency department.

## Introduction

Central venous catheter (CVC) placement is a commonly performed procedure for critically ill patients in emergency department. It is indicated for different purposes including central venous pressure (CVP) monitoring, infusion of vasoactive agents, administration of blood products, parenteral nutrition, and etc. CVC placement requires training and experience and is not risk-free for patients, even when performed by skilled professionals. 

CVC misplacement is a well-known technical error that has been described in approximately 7% of cases and causes serious complications such as haematoma, pneumothorax, catheter wedging, erosion or perforation of vessel walls, local venous thrombosis, catheter dysfunction, and cranial retrograde injection (1, 2).

Several techniques have been proposed to detect CVC placement. Most of the techniques require equipment such as fluoroscopy (3), electrocardiography (4), or invasive pressure monitoring (5), which may not be available in all hospitals. Misplacement of CVC is often diagnosed on post procedure chest X-ray (CXR) (6, 7). Confirmation of CVC placement with CXR is time-consuming and will expose the patient to radiation (8, 9). 

Saline flush test is another way for detection of CVC misplacement (10). Rath et al. showed that saline flush test had 100% sensitivity and specificity to detect misplacement of subclavian vein catheter into ipsilateral internal jugular vein (11). While Weekes et al. showed that rapid atrial swirl sign for subclavian and internal jugular CVC placement yielded 75% sensitivity and 100% specificity (12). Based on above mentioned discrepancy, this study was designed to compare the diagnostic accuracy of saline flush with CXR in confirmation of above-the-diaphragm CVC placement.

## Methods

***Study design and setting***

This diagnostic accuracy study was conducted to compare two different methods of CVC confirmation in Emergency Departments of Alzahra and Kashani Hospitals, Isfahan, Iran, from January 2015 to March 2016. The study was approved by the ethics committee of Isfahan University of Medical Sciences and authors adhered to the principles introduced in declaration of Helsinki during the study period. 

***Participants:***

Adult patients in need of internal jugular or subclavian CVC in Emergency Department based on clinical indications such as CVP monitoring, infusion of vasoactive agents, administration of blood products, and etc. were enrolled. Indication of CVC had been diagnosed by the Emergency Medicine specialist in charge of the patient’s treatment. Patients with infection on puncture site, thrombosis of the target vein, right atrial mass, clavicle or proximal rib fractures, significant high-acuity traumatic conditions, pre-existing internal jugular catheter or indwelling subclavian device, SVC syndrome, inability to obtain adequate subcostal or apical four-chamber images were excluded.

***Procedure:***

The neck and insertion region of target vein were cleaned with antiseptic solutions and isolated by sterile drapes when patient was in the supine position. An 18 Gauge introducer needle was inserted at the predetermined puncture point. After aspirating free flow of venous blood, J-tipped guide wire was inserted and the introducer needle was removed. Skin and subcutaneous tissue overlying the guide wire were dilated then CVC was railroaded over the guide wire 11–14 cm into the vein.

**Figure 1:**
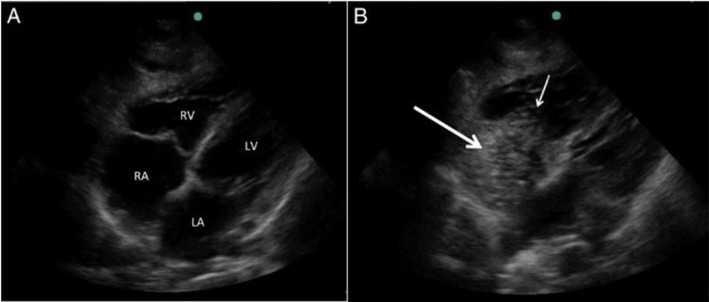
(A) Ultrasound view of the heart in the subxiphoid window during saline flush of distal central venous catheter (CVC) port. The right atrium (RA) and right ventricle (RV) are noted at time 0, at start of saline flush. (B) Microbubbles visible within the RA (bold arrow) and leading into the RV (small arrow) during the saline flush at the 1-second mark indicating CVC tip in correct position in the SVC.

A 10-mL sterile saline flush of the distal CVC port was performed simultaneously until the initiation of prospective recording of a 6-second digital video clip of either a standard subcostal or an apical four-chamber transthoracic cardiac window (figure 1) with ultrasound device (FUKUDA DENSHI UF-750XT class I). 

Ultrasonography was performed by one Emergency Medicine resident who had undergone 6 hours of training by a radiologist using pictures and videos.

Proper placement of CVC tip was defined as seeing the saline swirl entering the right atrium within 2 seconds of starting the saline flush. The 2-second cut-off was chosen based on a previous investigation by Vezzani et al. (9) that used this cut-off for seeing the entrance of agitated saline into the right atrium via echocardiography. By using contrast-enhanced echo to visualize the saline flush, they found that sensitivity and specificity of this method for detecting catheter misplacement were 96% and 93%, respectively (9), which inspired us to use this cut-off. When in doubt, saline flush was repeated during real-time monitoring of the right atrium.

Both onset and appearance of the turbulence were subjectively rated at the bedside by one emergency physician. Post-procedural CXRs were obtained in anterior-posterior view using a portable x ray machine. All CXRs were interpreted regarding the position of CVC tip and probable complications such as hemothorax, pneumothorax or pneumo-mediastinum, by the in-charge emergency medicine specialist. Then, chest computed tomography (CT) was performed, as the gold standard test, in order to confirm CVC placement in all cases. Chest CTs were interpreted by another emergency medicine specialist who was blinded to the patients’ data. All CXR and chest CT interpretations were double checked by a radiologist.

**Data analysis**

Statistical analysis was performed using SPSS version 21 and STATA 11 software and data were reported as mean ± standard deviation or frequency and percentage. Paired samples T test was used to compare the mean duration of saline flush and CXR methods for CVC confirmation. Sensitivity, specificity, positive and negative predictive values, positive and negative likelihood ratios as well as area under the ROC curve were calculated for each method considering CT scan as the reference test and using VassarStats medical calculator. Area under the ROC curves of the two methods was compared using chi-square test. All data were presented with 95% confidence intervals and two-tailed P<0.05 was considered significant. Accuracy of 0.90-0.100 was considered as excellent, 0.80-0.90 as good, 0.70-0.80 as fair, 0.60-0.70 as poor, and 0.50-.60 as fail.

## Results

***Baseline characteristics***

**Figure 2:**
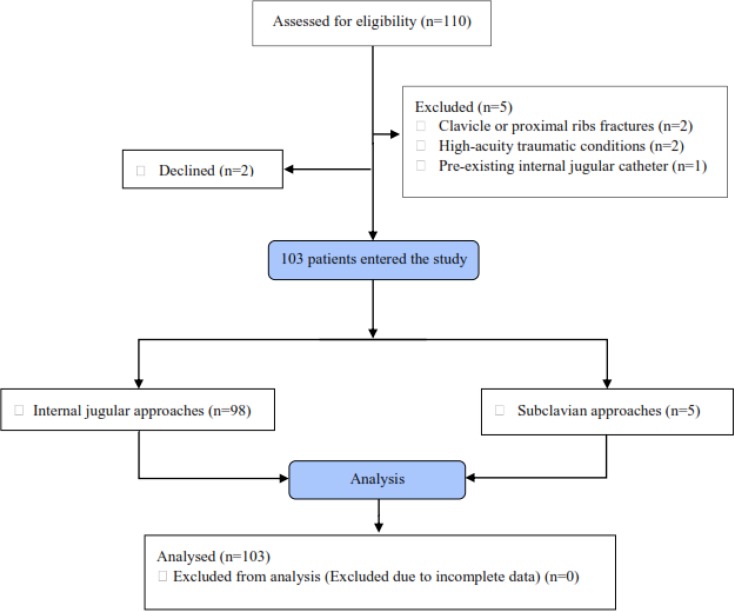
Study flowchart (CONSORT format)

103 cases of CVC were studied (95.1% jugular vein and 4.9% subclavian vein). Study flowchart is shown in figure 2. The mean age of studied patients was 57.18 ± 9.3 (35 -80) years (52.4% male). 

The mean duration of procedure was 2.5±1.24 minutes in saline flush method and 32.11±5.52 minutes in CXR method (P<0.001(. Serious complications such as hemothorax and pneumothorax were not observed in any cases.

***Accuracy of methods***

**Figure 3:**
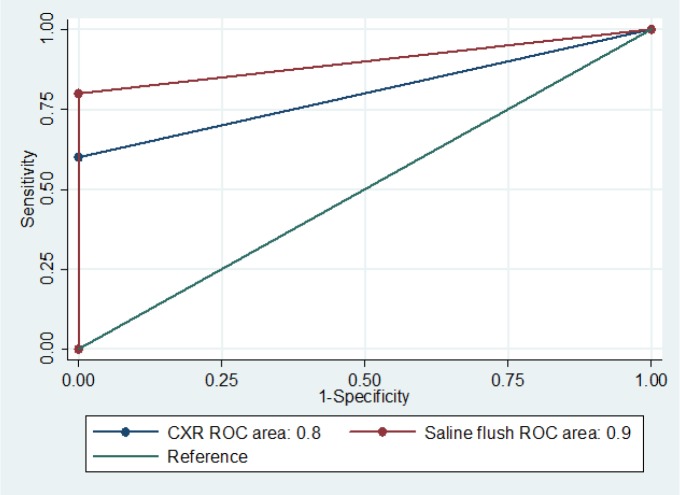
The area under the receiver operating characteristic (ROC) curves of saline flush and chest X-ray (CXR) in confirmation of central venous catheter (CVC) placement (p = 0.317).

In CT scan, 5 (4.9%) patients had CVC misplacement (3 cases of internal jugular approach and 2 cases of subclavian approach). Saline flush method confirmed 4 of the 5 (80%) mentioned cases and CXR confirmed 3 of the 5 (60%) misplacement cases. Table 1 and figure 3 compare the screening performance characteristics of the two methods considering the CT scan findings as the standard. 

The area under the ROC curves for saline method and CXR in confirmation of CVC placement were 0.90 (95%CI: 0.70 – 0.100) and 0.80 (95%CI: 0.55 – 0.100), respectively (p = 0.317).

## Discussion

Based on the findings of the present study, the overall accuracies of saline flush and CXR in CVC placement confirmation were in the same range (good: 0.80-0.90). However, saline flush method took significantly less time and had a higher specificity in this regard. Therefore, it seems that saline flush method could be considered as a safe, rapid, and accurate bedside method of CVC placement confirmation in emergency department.

**Table 1:** Screening performance characteristics of saline flush and chest X-ray (CXR) in confirmation of central venous catheter placement

**Characteristics**	**Saline flash**	**CXR**
**True positive**	4	3
**True negative**	98	98
**False positive **	0	0
**False negative**	1	2
**Sensitivity**	80 (29.87 – 98.94)	60 (17.04 – 92.74)
**Specificity**	100 (95.29 – 100)	100 (95.29 – 100)
**Positive predictive value**	100 (30.99 – 100)	100 (30.99 – 100)
**Negative predictive value**	98.98 (93.69 – 99.94)	98 (92.26 – 99.65)
**Positive likelihood ratio**	Infinity (NaN – Infinity)	Infinity (NaN – Infinity)
**Negative likelihood ratio**	0.01 (0.001 – 0.07)	0.02 (0.005 – 0.08)

Data were presented with 95% confidence interval; **NaN:** Calculation cannot be performed because the values entered include one or more instances of zero.

In Gekle R et al. study the mean confirmation time of CVC placement was 8.80 minutes with ultrasonography and 45.78 minutes with CXR (13). Moreover, Duran-Gehring PE et al. showed the mean total ultrasonography time was 5.0 minutes compared to 28.2 minutes for CXR (14). Maury et al. showed the mentioned times to be 6.8±3.5 and 80.3±66.7 minutes, respectively (8). 

All of these results are in line with our findings. On the other hand, studies have clarified that CXR is associated with radiation exposure and additional costs (8, 9).

However, using ultrasonography for confirmation of CVC placement has some limitations, including being dependent on operator performance. In evaluation of 83 CVC placements with ultrasonography, Matsushima and Frankel showed its poor sensitivity (50%) (15). 

Weekes et al. (12) compared contrast-enhanced ultrasonography alone with CXR for confirmation of proper catheter tip placement in 135 emergency department patients, and reported 75% sensitivity and 100% specificity for this method.

It has been suggested that performing CXR is not necessary when CVC placement is uncomplicated (16, 17). In one study it was concluded that when right internal jugular CVC placement was uncomplicated CXR could be safely skipped (18). Therefore, according to proven benefits of saline flush tests in detecting CVC placement compared to CXR (lower time, higher sensitivity, radiation free), it is a more effective method. However, saline flush test requires equipment such as ultrasonography and a person who is expert in performing the test. Therefore, teaching this method to emergency physicians should be considered in educational courses.

Limitation

Since the studied patients were all critically ill, all the CXRs have been performed in the anterior-posterior view, which can affect its accuracy. Duration of training for the ultrasonography performer and his/her skill in interpretation and performing ultrasonography are also among other limitations that can affect the accuracy of this method to a great extent.

## Conclusion

Based on the findings of the present study, the overall accuracies of saline flush and CXR in CVC placement conformation were in same range (good: 0.80-0.90) without any significant difference. However, saline flush method took significantly less time and had higher specificity in this regard. Therefore, it seems that saline flush method could be considered as a safe, rapid, and accurate bedside method of CVC placement confirmation in emergency department.
